# Inheritance, QTLs, and Candidate Genes of Lint Percentage in Upland Cotton

**DOI:** 10.3389/fgene.2022.855574

**Published:** 2022-03-31

**Authors:** Hao Niu, Qun Ge, Haihong Shang, Youlu Yuan

**Affiliations:** ^1^ State Key Laboratory of Cotton Biology, Key Laboratory of Biological and Genetic Breeding of Cotton, The Ministry of Agriculture, Institute of Cotton Research, Chinese Academy of Agricultural Sciences, Anyang, China; ^2^ Zhengzhou Research Base, State Key Laboratory of Cotton Biology, Zhengzhou University, Zhengzhou, China

**Keywords:** upland cotton (*Gossypium hirsutum* L.), lint percentage, inheritance, quantitative trait locus, gene

## Abstract

Cotton (*Gossypium* spp.) is an important natural fiber plant. Lint percentage (LP) is one of the most important determinants of cotton yield and is a typical quantitative trait with high variation and heritability. Many cotton LP genetic linkages and association maps have been reported. This work summarizes the inheritance, quantitative trait loci (QTLs), and candidate genes of LP to facilitate LP genetic study and molecular breeding. More than 1439 QTLs controlling LP have been reported. Excluding replicate QTLs, 417 unique QTLs have been identified on 26 chromosomes, including 243 QTLs identified at LOD >3. More than 60 are stable, major effective QTLs that can be used in marker-assisted selection (MAS). More than 90 candidate genes for LP have been reported. These genes encode MYB, HOX, NET, and other proteins, and most are preferentially expressed during fiber initiation and elongation. A putative molecular regulatory model of LP was constructed and provides the foundation for the genetic study and molecular breeding of LP.

## Introduction

Cotton (*Gossypium* spp.) is an important natural fiber plant. The genus *Gossypium* consists of 52 wild and cultivated species, including 6 allotetraploid species and 46 diploids (Wendel and Cronn, 2003; Chen et al., 2016; Wang et al., 2021). Cultivated species include two diploid species (2n = 2x = 26), *Gossypium herbaceum* L. (A_1_) and *Gossypium arboreum* L. (A_2_); and two tetraploid species (2n = 4x = 52), upland cotton [*Gossypium hirsutum* L. (AD_1_)] and sea island cotton [*Gossypium barbadense* L. (AD_2_)]. Among the cultivated species, upland cotton is the most widely cultivated, accounting for approximately 95% of worldwide cotton production (Chen et al., 2007; Imran et al., 2012). Cotton yield is typically influenced by multiple complex quantitative traits, including boll number per plant (BN), single boll weight (BW), lint percentage (LP), seed index (SI), and lint index (LI) (Qin et al., 2008; Yu et al., 2016). Of these, LP is a key contributor to lint yield and a critical economic index for cotton cultivars, and measurement of LP is easy during cotton breeding (Tang and Xiao, 2013; Su et al., 2016; Song et al., 2019).

Cotton breeding is performed to achieve simultaneous improvement of cotton yield and fiber quality (Wang et al., 2011). However, LP is positively or negatively correlated with other yield and fiber quality components (Kong et al., 2000; Ulloa and Meredith, 2000; Wang et al., 2007; Imran et al., 2012; Tang and Xiao, 2013; Wang et al., 2014; Carvalho et al., 2015; Chen et al., 2019; Zhu et al., 2021). Selecting for LP alone to increase lint yield reduced boll and seed mass, but also fiber length and fiber strength (Wang et al., 2011). The negative correlation between these traits and the reduction in a cotton cultivar due to the introduction of deleterious genes along with the beneficial ones make it challenging to synchronously improve fiber quality and lint yield. Thus, effective cotton breeding requires characterization of the inheritance, quantitative trait loci (QTLs), candidate genes, molecular regulatory networks of LP, and other yield and fiber quality components. By using big segregating populations and whole genome selection techniques, it may be possible to break tight linkages of QTLs with the opposite genetic effects.

Genetic study, germplasm resource enhancement, and breeding of upland cotton are very important for efforts to improve cotton production. Abundant cotton germplasm resources with high genetic diversity can be used to improve upland cotton, and LP can be used as an indicator of genetic diversity. There have been recent advances in the molecular and genetic basis of cotton LP, however, the genetic basis of cotton LP remains incompletely understood. To provide detailed genetic information related to cotton LP for future cotton lint yield studies and breeding, we comprehensively summarized the inheritance, QTLs, and related candidate genes of cotton LP. We reanalyzed available data and mined the QTLs and candidate LP genes, screened the stable and most effective QTLs for use in marker-assisted selection (MAS), identified the key biological pathways regulating LP, and outlined a molecular regulatory network for cotton LP development. These results can guide cotton yield improvement.

## Inheritance of Cotton Lint Percentage

### LP is a Typical Quantitative Trait Controlled by Major Genes Plus Polygenes

Cotton yield per unit area includes four major components, plant number per unit area, boll number per plant, single boll weight, and LP (Yu et al., 2016). LP (also known as ginning out-turn) is the ratio of lint weight to seed cotton weight [or lint index/(lint index + seed index)] as a percentage. LP is determined by fiber number, fiber length, fiber diameter, and the weight of seed on which the fibers are grown. LP is a critical criterion for cotton cultivars, and it influences the purchase price of the seed cotton. Historic data of Huang-Huai Regional Trials in China from 1973 to 1996 reported an increase in yield from 1200 kg·hm^−2^–1425 kg·hm^−2^ that was explained by improvements of LP, which increased 5.5% (Jiang et al., 2000). Obviously, LP is a key selection trait for cotton yield breeding.

The phenotypes of complex traits are often determined by the combined actions of multiple genes and environmental factors (Mackay et al., 2009). Cotton yield and its components, BN, BW, LP, SI, and LI, are inherited quantitatively and are genetically related to each other (Wang et al., 2007; Qin et al., 2009). Of these components, the heritability of LP is the highest (49%; Badigannavar and Myers, 2015), and LP correlates with seed cotton yield, lint yield and other yield components at different strengths (Wang et al., 2014; Chen et al., 2019; Zhu et al., 2021). LP is a typical quantitative trait of cotton cultivars, except in fuzz-less and lint-less mutants where it is considered a qualitative trait (An et al., 2010).

We can choose appropriate breeding methods to improve cotton yield when there are good genetic models of yield-related traits. Studies of the inheritance of cotton LP began in the 1970s in China. First, simple crosses were performed between high and low LP cultivars, and then the LP of the offspring was evaluated. Overall, the inheritance of LP was not clear. In the 1990s, the model of major genes plus polygenes became more widely studied (Mo, 1993), allowing the later establishment of a mixed genetic model of major genes plus polygenes. The model assumed that the studied traits were diploid inherited, and lacked female effects, interaction, and linkage among major genes and polygenes (Gai et al., 2003). A genetic model to simultaneously analyze genetic effects of nuclear, cytoplasm, and nuclear-cytoplasmic interaction (NCI) and genotype by environment (GE) interactions for quantitative traits was subsequently developed (Han et al., 2007). These methods have been applied in genetic studies on cotton yield related traits, including LP (Du et al., 1999; Yuan et al., 2002; Zeng and Wu, 2012; Xia et al., 2014; Li B et al., 2016; Gong et al., 2019).

Lint yield and yield components are mainly controlled by genetic effects, i.e., additive and dominant variance account for ≥66% of the total phenotypic variance (Zeng and Wu, 2012). LP has the highest additive variance as a proportion of the total phenotypic variance (Zeng and Wu, 2012). LP is controlled mainly by genetic effects and is less affected by GE interaction effects (Yuan et al., 2002; Han et al., 2007). The variances of additive effect (VA), cytoplasmic effect (VC), and the dominant nuclear-cytoplasmic interaction effect (DC) interaction variance (VDC) are significant for LP (Han et al., 2007). LP shows great genetic variability. Other quality parameters have high heritability values (up to 90%), while LP exhibits moderate heritability (49%) (Badigannavar and Myers, 2015).

Segregation analysis of the mixed genetic model of major genes plus polygenes was used to identify the major genes for cotton yield-related traits using six generations of P_1_, P_2_, F_1_, B_1_, B_2_, and F_2_ generated from a cross of Baimian1 × TM-1 (TexasMarker-1) (Jia et al., 2014). One major gene of LP was detected, with positive complete dominance, and an additive effect value equal to the dominant effect value (0.512) (Xia et al., 2014). A major QTL, *qLP-3b*(F_2_)/*qLP-3*(F_2:3_), of LP was detected in both F_2_ and F_2:3_ using QTL mapping, with additive effect values (1.86 and 1.49, respectively) slightly smaller than the dominant effect values (1.95 and 1.51, respectively), thus displaying a dominant effect (Xia et al., 2014).

We studied LP using a female parent sGK Zhong156, male parent 901-001, and their 250 introgression lines (ILs) and found that LP is controlled by two to four pairs of major genes or two pairs of major genes plus polygenes. The heritability of major genes ranged from 1.26 to 83.13%, the heritability of polygenes ranged from 27.35 to 90.83%, and the heritability of major genes plus polygenes ranged from 92.00 to 99.35% (Gong et al., 2019).

Overall, the contributions of V_G_ and V_GE_ to total genetic variances of LP are about 73.62 and 26.38% (Han et al., 2007). LP is determined by additive effects (65.4%) (Yuan et al., 2002; Saha et al., 2006, 2010; Liu et al., 2012; Li C et al., 2016), non-additive effects (Liang et al., 2012), dominant effects (16.8%) (Yuan et al., 2002; Saha et al., 2006; Xia et al., 2014), cytoplasmic effects, and dominant nuclear-cytoplasmic interaction effect variance (Saha et al., 2006; Han et al., 2007; Liu et al., 2012; Li et al., 2017). The results indicated that LP is mainly controlled by major genes plus polygenes with additive and dominant effects (Cheng et al., 1998; Du et al., 1999; Xia et al., 2014; Gong et al., 2019).

### LP Correlates With Other Components of Yield and Fiber Quality

Many agronomic traits are correlated due to linkage, pleiotropy, or the correlated traits are components of a more complex variable (Ulloa et al., 2005). LP is positively or negatively correlated with other components. Generally, LP has significantly negative correlation with seeds per boll, seed index, seed cotton yield, fiber length (FL), and fiber strength; and is positively correlated with boll number per plant, lint per seed, lint index, and lint yield (Kong et al., 2000; Ulloa and Meredith, 2000; Wang et al., 2007; Imran et al., 2012; Tang and Xiao, 2013; Carvalho et al., 2015; Zhu et al., 2021).

### Germplasm Resources and Enhancement of High LP Cotton

Excellent germplasm resources are required for the successful breeding of cotton cultivars (Zeng and Wu, 2012). Based on introducing foreign cotton cultivars, three cultivar substitutions happened in China from the 1920s, first, Jinzimian, Tuozimian, and Longzimian from the United States substituted some Asiatic cotton cultivars planted in north China in 1920s; second, Sizimian4, Dezimian531, and Daizimian14 substituted the Asiatic cotton cultivars planted in a large area of China in the 1930s–1940s; third, Daizimian15, Sizimian2B, and Sizimian5A substituted the Asiatic cotton cultivars planted for a long period, and the degenerated foreign upland cultivars in China in the 1950s (Yin et al., 2002).

Chinese researchers traveled to Mexico, the United States, France, Australia, and other countries to explore and collect cotton germplasm resources from the 1970s to 1980s, with more than 4800 cultivars, lines, wild species, semi-wild species, and special lines with genetic markers collected by 1984 (Gao et al., 2021). A total of 8868 accessions were collected and preserved by 2020, including 7362 *G. hirsutum*, 350 *G. hirsutum* wild races, 633 *G. barbadence*, 433 *G. arbareum*, 18 *G. herbaceous*, and 32 wild species, making the cotton collection in China the fourth largest in the world (Du et al., 2012). Many of these cotton resources have been characterized. For example, 28 cotton lines with high LP (43–47%) were screened by Li and Wang (1992), and 8 high LP (> 42%) lines were screened from former Soviet Union germplasm resources by Liu et al. (1998). Cultivars introduced from the former Soviet Union (108Ф, C1470, 611B, and KK1543), the United States, and Africa (DPL15, STV2B, and UGDM) were applied for modern cultivar improvement in Xinjiang, China (Han et al., 2020). Using foreign germplasm resources, many high LP (> 43%) cultivars/lines have been bred in China.

In addition to traditional methods of plant cultivar improvement, such as intraspecific crosses and physical and chemical mutagenesis (Gai, 1997; Hulse-Kemp et al., 2015), the introduction of elite alien genes or chromosome fragments by interspecific crosses is a main approach for cotton germplasm enhancement, especially for LP. Upland cotton has a high lint yield but undesirable fiber quality, sea island cotton has superior fiber quality but lower lint yield. Therefore, significant efforts have been made worldwide to combine the desirable lint yield of *G. hirsutum* with the superior fiber quality of *G. barbadense* by interspecific hybridization. Many germplasm resources with improved fiber quality containing chromosomal introgressions from *G. barbadense* have been constructed (Saha et al., 2006; Saha et al., 2010; Zhu et al., 2010; Wang et al., 2011; Zhu et al., 2017; Chen et al., 2018; Li et al., 2019) (Supplementary Table S1). Compared to *G. barbadense*, the superior introgression lines (IL) have fiber quality more similar to that of *G. barbadense* and are usually more convenient to use for upland cotton improvement (Wang et al., 2011).

Artificial selection efforts have reduced genetic diversity but significantly increased LP. A cotton variation map of an intact breeding pedigree including 7 elite and 19 backbone parents was constructed (Ma et al., 2019). The 26 pedigree accessions were subjected to strong artificial selection during domestication and have reduced genetic diversity but stronger linkage disequilibrium and higher extents of selective sweeps. The elite parents acquired significantly improved agronomic traits, with an especially pronounced increase in LP. Although there is a negative correlation between LP and FL in long fiber materials (Carvalho et al., 2015), two cultivars and one advanced line have the highest LP and FL as well as high productivity: FM 993, FM 910, and CNPA MT 04 2088, suggesting these materials can be used to increase LP and FL values (Santos et al., 2017).

A set of chromosome segment introgression lines (CSILs) were developed by crossing the recipient parent TM-1 and the donor parent Hai7124. TM-1 is a genetic *G. hirsutum* standard accession developed in the United States, and Hai7124 is an early, high-yielding *G. barbadense* accession developed in China (Song and Zhang, 2009). This set of CSILs has been used to explore the genetic basis of heterosis for interspecific hybrids (Guo et al., 2013). A set of 14 chromosome substitutions (CS-B) with specific chromosomes or chromosome arms from *G. barbadense* substituted into *G. hirsutum* and chromosome-specific F_2_ families has been reported, and additive and dominant effects are significant for LP (Saha et al., 2006). Another set of 17 CS-B lines (2n = 52) were reported that contain *G. barbadense* doubled-haploid line “3-79” germplasm systematically introgressed into “TM-1”. TM-1 yield is much greater than 3-79, but cotton from 3-79 has superior fiber properties. CS-B14sh, 17, 22Lo, and 25 produce positive homozygous dominant effects on lint yield (Saha et al., 2010). A CSSL population was developed via crossing and backcrossing of *G. hirsutum* and *G. barbadense* by PCR-based MAS (Zhu D et al., 2020). By whole genome re-sequencing, 11,653,661 high-quality SNPs were identified with 1211 recombination chromosome introgression segments from *G. barbadense*. Six QTLs for LP were identified that have negative alleles from *G. hirsutum* background.

Extensive efforts have generated successful intraspecific crosses of *G. hirsutum*, and interspecific crosses between *G. hirsutum* and *G. barbadense*. Many interspecific ILs or CSILs have been developed and studied (Shi et al., 2015; Li et al., 2019), and some ILs have been identified with positive effects that increase LP, such as the IL008 lines (Zhu et al., 2017). Many advanced new cotton germplasm resources and cultivars have been bred, such as C^24^ (LP = 48–50%; Wang and Xiao, 1996), IL-10-1 (LP > 37%; Ma et al., 2012), S26-1 (LP > 46%), Malan (LP > 46%), Xingtai6871 (LP > 46%), Shan815 (LP > 45%), Kang35 (LP > 45%) (Li and Wang, 1992), Lumianyan22 (Chen et al., 2018), and Sumian16 and Simian3 (both LP > 43%; Zhang Z et al., 2005). These enhanced cotton germplasm resources can be used for LP breeding in upland cotton.

### The Heterosis of LP

Utilization of heterosis has greatly improved the productivity of many crops worldwide, including cotton. Hybrid cotton varieties exhibit strong heterosis that confers high fiber yields (Ma et al., 2019). Understanding the potential molecular mechanism underlying how hybridization produces superior yield in upland cotton is critical for effective breeding (Shahzad et al., 2020). Although the role of loci with over-dominant effects continues to be discussed by biologists, the genetic basis of heterosis has not yet been determined. Genetic models are important, as heterosis is a nonlinear effect from multiple heterozygous gene combinations (Gai et al., 2007). Trait phenotype and heterosis may be controlled by different sets of loci and many genetic factors likely function together to yield heterotic output (Shahzad et al., 2020). All three forms of genetic effects, additive, over-dominant, and epistasis, and environmental interaction contribute to the heterosis of yield and its components in upland cotton, with over-dominance and epistasis the most important (Guo et al., 2013; Li et al., 2018).

A study with 286 upland cotton cultivars found that female parent groups based on genetic distance (GD) and F_1_ performance significantly differ in LP. The correlations between GD and F_1_ performance, mid-parent heterosis (MPH), and best parent heterosis (BPH) are significant for LP (Geng et al., 2021). Fourteen putative candidate genes were identified as associated with heterosis of LP (Sarfraz et al., 2021). A study with CP-15/2, NIAB Krishma, CIM-482, MS-39, and S-12 found greater specific combining ability (SCA) variance than general combining ability (GCA) variance for LP (0.470), indicating the predominance of non-additive genes (Imran et al., 2012). Dominance plays an important role in the genetic basis of heterosis in Xiangzamian2, and non-additive inheritance is also an important genetic mode for LP (Liu et al., 2012).

Studies on the interspecific heterosis of *G. hirsutum* × *G. barbadense* were performed starting in the 1960s in China (Diao and Huang, 1961; Hua et al., 1963). Although many traits exhibit heterosis such as boll setting, fiber quality, photosynthesis, and drought and disease resistance, some hybrid cultivars suffer from severe defects that preclude planting in many areas, with characteristics such as small bolls, low LP, and low yield (Chen et al., 2002). Breeding chromosome segment substitution lines (CSSLs) is a main strategy to utilize excellent genes from *G. barbadense* and partial interspecific heterosis in upland cotton.

Studies with CSSLs and their 50 F_1_ hybrids found that yield and yield components are controlled by combined additive and dominant effects, and LP is mainly controlled by additive effects. Significant positive MPD was detected for all yield traits, and significant positive BPH was detected for all yield traits except LP (Shen et al., 2006). A proposed model was studied in *G. hirsutum* introgression lines (ILs) harboring a segment of *G. barbadense*. These ILs contained 396 QTLs for 11 yield and other traits. The yield group has significantly higher over-dominant QTL ratios, with 16 over-dominant QTLs for 5 yield-related traits consistently detected during 5 cotton growing seasons, one of which is LP. Overdominance is the major genetic basis of lint yield heterosis in interspecific hybrids between *G. barbadense* and *G. hirsutum* (Tian et al., 2019).

Over-dominance is a major factor for yield heterotic output, so genetic diversity is especially important. Genitors with short GD share many alleles, and there are fewer complementary genes in heterozygotes. The genetic diversity among 16 cotton cultivars was studied. Variables with low magnitude, redundancy, and non-stability are not ideal for genetic diversity study, but LP is the trait that most contributes to genetic diversity at 31.3% (Santos et al., 2017).

Although the genetic basis of heterosis has not been resolved in cotton, the available data indicate that LP is a significant contributor to genetic diversity (Santos et al., 2017). SCA variance is greater than GCA variance for LP, showing the predominant contribution of non-additive genes (Imran et al., 2012). Overdominance is the major genetic basis of lint yield heterosis in interspecific hybrids of *G. barbadense* and *G. hirsutum*, including LP (Tian et al., 2019), and LP has significant negative heterosis value (Yuan et al., 2002; Shen et al., 2007). Additive and over-dominant effects have been found for QTLs of LP (Liu et al., 2011; 2012). At the single-locus level, the genetic bases of heterosis vary in different populations. In conclusion, additive effects, over-dominant effects, epistasis, and environmental interactions all contribute to the heterosis of yield and its components in upland cotton, with over-dominance and epistasis the most important (Li et al., 2018).

## QTLs of Cotton Lint Percentage

### High-Density Genetic Maps of Cotton LP

A QTL is a “gene” controlling a certain quantitative trait. Although where it is and what it encodes may be unknown, the term QTL theoretically links a gene and a trait. QTLs can be detected and located by genetic linkage analysis and QTL mapping relying on a similar set of genetic assumptions. Genetic linkage analysis can detect both major gene and polygene effects, does not need a linkage map and provides no information about the location of the QTL. QTL mapping can detect and locate a possible QTL if a linkage map is available (Gai et al., 2007). Significant advances in QTL mapping occurred in the 1990s, enabling the study of individual QTLs and interaction between QTLs for heterosis, providing a powerful tool to identify the genetic bases of yield and yield components (Liu et al., 2012; Li et al., 2018). QTL mapping using populations segregating for mutants can increase the sensitivity of this method due to the enlarged quantitative variation scale by the expression of qualitative mutant genes (An et al., 2010). In the discovery of major genes or major QTLs, segregation analysis and QTL mapping are mutually complementary methods that can verify results.

The first cotton linkage map was reported in 1985, with 11 linked groups located on chromosomes, and mapping of some mutant genes, including two LP genes (Endrizzi et al., 1985). Currently, more than 31 genetic linkage maps have been reported in upland cotton (Supplementary Table S1). Crosses including intra-species of *G. hirsutum*, inter-species of *G. hirsutum* and *G. barbadense*, populations of immortalized F_2_ and F_2:3_, RIL, backcross inbred lines (BIL), and immortalized backcross populations (DHBCF1s and JMBCF1s). Various molecular markers such as simple sequence repeats (SSR), expressed sequence tag-SSR (EST-SSR), fragment length polymorphism (RFLP), random amplified polymorphic DNA (RAPD), amplified fragment length polymorphism (AFLP), chip-SNP, and whole genome re-sequencing have been employed to make genetic linkage maps of cotton. The density of the genetic maps has increased, and the marker span has reached 5115.16 cM, with an average marker interval that is less than 1 cM 0.92, 0.72, and 0.5 cM (Blenda et al., 2012; Shi et al., 2015; Diouf et al., 2018; Gu et al., 2020). We have constructed a consensus genetic linkage map covering the whole genome with 8295 markers (458 SSR, 5521 SLAF-SNP, and 2316 chip-SNP), spanning 5197.17 cM, with 2384.94 cM for the A_t_ subgenome and 2812.23 cM for the D_t_ subgenome. The average interval between adjacent markers is 0.88 cM (Zhang K et al., 2019).

With the development of techniques related to genetic mapping, the coverage and accuracy of the maps are constantly increasing, enabling discovery of more QTLs. High density maps make it possible to clone candidate genes of major effective QTLs, including LP. As more QTLs are discovered, the number of high-quality QTLs for LP with closely linked markers has increased, leading to the discovery of more effective candidate genes. Thus, these improved techniques have greatly facilitated molecular breeding and cotton LP improvement efforts. Some genetic and association maps reported in cotton, including the numbers of the identified QTLs for LP, are listed in Supplementary Table S1.

#### High-Density Genetic Maps of *G. hirsutum*

More than 17 crosses or populations of upland cotton have been used to construct genetic maps (Supplementary Table S1), such as crosses of Yumian1 × T586 (Zhang P et al., 2005; Wan et al., 2007; Liu D et al., 2015), Yumian1 × Zhongmiansuo35 (Chen et al., 2008), NC05AZ06 × NC11-2091 (Zhang Z et al., 2019), DH962 × Jimian5 (Lin et al., 2009a, b; Wang et al., 2016), Zhongmiansuo12 (ZMS12) × 8891 (Wang et al., 2007), (Simian3 × Sumian12) × (Zhong4133 × 8891) (Qin et al., 2015), Baimian1 × TM-1 (Wang et al., 2014; Xia et al., 2014), Xiangzamian2 (Liu et al., 2011; Liu et al., 2012), HS46 × MARCABUCAG8US-1-88 (Wu et al., 2009; Li C et al., 2016), and CCRI35 × Nan Dan Ba Di Da Hua (NH) (Diouf et al., 2018). With more advanced studies, the densities of the genetic maps have increased significantly. For example, a high-density genetic map was drawn using 588 F_7_ RILs derived from the cross of Nongdamian13 × Nongda601 by a whole genome re-sequencing strategy. This high-density bin linkage map contains 6187 bin markers spanning 4478.98 cM with an average distance of 0.72 cM. Fifty-eight individual QTLs and 25 QTL clusters harboring 94 QTLs, and 119 previously undescribed QTLs controlling 13 fiber quality and yield traits across 8 environments were identified, including 17 QTLs for LP (Gu et al., 2020).

#### High-Density Genetic Maps of *G. hirsutum* × *G. barbadense*

Cotton genetic maps have been constructed with interspecific crosses of *G. hirsutum* × *G. barbadense* (Supplementary Table S1) (Li et al., 2009; Blenda et al., 2012; Yu et al., 2013; Shi et al., 2015). For example, a high-density SSR genetic linkage map has been drawn using a BC_1_F_1_ population developed from a cross of *G. hirsutum* × *G. barbadense*. The map comprises 2292 loci and covers 5115.16 cM of the cotton AD genome, with an average marker interval of 2.23 cM. For 1577 common loci on this map, 90.36% agree well with the marker order on the D genome sequence genetic map. Twenty-six QTLs for LP were identified on 9 chromosomes, and 50% of the QTLs are from *G. barbadense* and increase LP by 1.07–2.41% (Shi et al., 2015).

#### High-Density Genetic Maps of QTLs From *G. barbadense*

Upland cotton CSILs with chromosome fragments from *G. barbadense* are ideal materials to identify new agronomic genes from sea island cotton, which can be identified using genetic maps (Supplementary Table S1) (Zhu et al., 2010; Wang et al., 2011; Guo et al., 2013; Said et al., 2015; Zhai et al., 2016; Zhu et al., 2017; Chen et al., 2018; Kong et al., 2018). For example, F_2_ and F_2:3_ populations derived from the cross of CSILs-IL-15-5 × IL-15-5-1 were used to map QTLs for LP and seed index with SSR markers. The SI and LP characteristics of 774 F_2_ individuals and F_2:3_ families were analyzed by composite interval mapping (CIM), and two QTLs were detected for LP: *qLP-15-1* in the F_2_ and F_2:3_ populations located between NAU3040 and JESPR152, with confident genetic distances of 5.40 and 3.20 cM, respectively, and *qLP-15-2*, mapped between NAU5302 and NAU2901 with confident genetic distance of 0.08 cM (Zhu et al., 2010). In this way, more than 107 QTLs for LP have been reported (Said et al., 2015) (Supplementary Table S1).

### QTLs Controlling Cotton LP

LP is controlled by many QTLs. Study of the F_2:3_ population derived from the cross of HS46 × MARCABUCAG8US-1-88 identified a single QTL that explains 15.45% of phenotypic variance of LP, with other QTLs that explain 10.03–15.46% (Diouf et al., 2018). Mapping where these genes reside on the chromosomes will facilitate breeding, especially if easily measured molecular markers are closely linked with the specific QTLs so they can be used in MAS (Shappley et al., 1998; Liu et al., 2014). The classical genetic method of studying quantitative traits is based on a linkage analysis of both parents, enabling the mapping of multiple QTLs responsible for LP using segregating populations from different parents (Dong et al., 2018). To date, many QTLs controlling LP have been reported (Table 1; Supplementary Table S2).

**TABLE 1:** The unique, tightly linked (LOD >3) and major effective QTLs for LP.

Chromosome No	Chr01	Chr02	Chr03	Chr04	Chr05	Chr06	Chr07	Chr08	Chr09
Unique QTLs	9	8	27	16	15	15	24	8	16
Tightly linked QTLs	3	5	19	10	8	12	14	5	6
Major QTLs	0	2	10	4	2	2	3	1	0

Chromosome No	Chr10	Chr11	Chr12	Chr13	Chr14	Chr15	Chr16	Chr17	Chr18
Unique QTLs	14	10	15	29	18	16	11	28	15
Tightly linked QTLs	7	6	8	20	12	10	8	19	4
Major QTLs	1	2	1	3	4	4	3	2	0

Chromosome No	Chr19	Chr20	Chr21	Chr22	Chr23	Chr24	Chr25	Chr26	Total
Unique QTLs	9	18	16	15	10	21	28	6	417
Tightly linked QTLs	2	10	11	10	7	11	13	3	243
Major QTLs	0	2	3	0	2	2	4	3	60

#### QTLs for LP in Upland Cotton

To identify QTLs for LP, mapping populations have been constructed using a high LP variety, Baimian1, and a low LP variety, TM-1. Six major effective QTLs (M-QTL) for LP were detected on chromosomes 3, 5, 19, and 26. Three of these, *qLP-3*(2010), *qLP-3*(2011), and *qLP-19*(2010), are significant M-QTLs (Wang et al., 2014). Four common QTLs for LP were discovered from the cross of Baimian1 × TM-1: *qLP-3b*(F_2_)/*qLP-3*(F_2:3_) and *qLP-19*b(F_2_)/*qLP-19*(F_2:3_) explaining 23.47 and 29.55% of phenotypic variation (Xia et al., 2014). Similarly, using a cross of Yumian1 × T586, four QTLs for LP were detected (Zhang P et al., 2005). Using 219 F_2_ individuals developed from a cross of TM-1 × T586, three LP QTLs were tagged and mapped on the A03 linkage group and chromosome 6 (Guo et al., 2006). The allele(s) originating from T586 of QTLs controlling LP can increase the trait phenotypic value (Wan et al., 2007). A recombinant inbred line (RIL) population including 188 RILs developed from 94 F_2_-derived families and their two parental lines, “HS46” and “MARCABUCAG8US-1-88”, were evaluated, and five QTLs responsible for 38.1% of the phenotypic variance for LP were mapped to chromosomes 3, 4, 9, 12, and 26. Chromosomes 3, 5, 12, 13, 14, 16, 20, and 26 harbor important QTLs for both yield and fiber quality. Chromosomes 3 and 26 are associated with QTLs for LP (Wu et al., 2009). Seventeen SSRs out of 304 markers tested from MGHES (EST-SSR), JESPR (genomic SSR), and TMB (BAC-derived SSR) collections show significant linkage associations with LP QTLs in a set of RILs segregating for LP (Abdurakhmonov et al., 2007). One dominant QTL for LP, *qLP43* from DH962, is near marker MGHES-75 (Lin et al., 2009a; b). Two QTLs for LP, *qLP-C13-1* and *qLP-C25-1* were detected from the high LP parent Lumianyan22, and explain 5.57–8.87% of phenotypic variation (Chen et al., 2018). The detailed information of QTLs for LP in upland cotton is summarized in Table 1 and Supplementary Tables S1, S2, S3, and S4.

#### QTLs for LP in Upland Cotton With Lintless Mutants

Using mapping populations developed from a *G. barbadense* line (fuzzy-linted) and a single fuzzless locus mutant (*G. hirsutum*, N_1_ or n_2_), considerable quantitative variation in lint fiber production superimposed on the discrete effects of fuzz mutants was observed and both loci mapped within the intervals of QTL for LP and lint index (Rong et al., 2005). These results were confirmed by QTL mapping for LP and fiber quality traits (micronaire, uniformity, and fiber elongation) using an n_2_ mutant derived population (Rong et al., 2007). Two F_2_ populations were studied from crosses of MD17, a fuzzless-lintless line containing three fuzzless loci, N_1_, n_2_ and a postulated n_3_, with line 181, fuzzless-linted and with FM966, a fuzzy-linted cultivar. QTLs for LP and LI explain a high percentage of the phenotypic variation (62.8–87.1%) and are probably on the same location on chromosome 26 with the common marker BNL3482-138 in both populations. The MD17 BNL3482-138 allele at this locus decreases both LP and LI (An et al., 2010) (Supplementary Table S1).

#### QTLs for LP From *G. barbadense*

Many QTLs for LP from *G. barbadense* have been identified. Eight QTLs for LP were discovered in an RIL population of *G. barbadense* (Fan et al., 2018). The F_2_ populations from two CSSLs MBI7455 and MBI7358 were quantified using SSR, and five LP QTLs were identified (Guo et al., 2015). Four CSILs, MBI7115, MBI7412, MBI7153, and MBI7346, were obtained by advanced backcrossing and continuous inbreeding of upland cotton variety CCRI45 and sea island cotton variety Hai1 and were used to construct double-cross segregating populations F_1_ and F_1:2_ through the following crosses [(MBI7115 × MBI7412) × (MBI7153 × MBI7346)]. The analysis revealed that three QTLs control LP (Kong et al., 2018). Four BC_1_F_1_ populations, E(E3), E(3E), (E3)E, and (3E)E, were developed by crossing sea island cotton 3-79 (“3”) and upland cotton “Emian22” (“E”), using “Emian22” as the recurrent parent. Using composite interval mapping, two LP QTLs were detected on a single chromosome (Dai et al., 2018). We previously developed a set of CSSLs using CCRI8 of *G. hirsutum* as the recipient parent and Pima90-53 of *G. barbadense* as the donor parent. We genotyped the BC_3_F_5_ generation of CSSLs with SSR markers, constructed a QTL map for fiber quality and yield traits, and identified the stable QTLs in three different environments (Baoding, Qingxian, and Luntai). Seven QTLs for LP were detected, and each QTL explained 2.03–19.38% of the phenotypic variation (Li et al., 2018).

### Stable QTLs for LP Can Be Used in MAS

DNA markers linked to agronomic traits increase the efficiency of breeding and significantly decrease the cost, time, and risk of subjective phenotypic assays. MAS can help by pyramiding favorable genes or alleles into a cultivar and breaking the negative relation between yield and quality (Zhang et al., 2011; Zhu et al., 2017). The application of MAS for the improvement of traits of interest depends on the availability of QTL information (QTL positions and effects) and flanking marker information (flanking marker positions and allele sizes) (Wu et al., 2009).

Most reported QTLs controlling LP have closely linked markers, enabling their use in MAS (Shappley et al., 1998). Some of this kind of QTLs have been reported, such as the stable *qLP-A10-1* from the cross of Zhongmiansuo12 (ZMS12) × 8891 (Wang et al., 2007); *qLP-15-1* and *qLP-15-2* in CSILs-IL-15 (Zhu et al., 2010); *qLP-2-1*, *qLP-2-2* and *qLP-4-2* from RILs derived from the cross of 0-153 × SGK9708 (Jia et al., 2011); *qLP-3b*(*F*_*2*_)*/qLP-3*(*F*_*2:3*_), detected from the cross of Baimian1 × TM-1 (Xia et al., 2014); six main-effect QTLs (M-QTL) on chromosomes 3, 5, 19, and 26; *qLP-3* and *qLP-19* (Wang et al., 2014); major QTL *qLP-3* from *G. mustelinum* (Chen et al., 2019); and three major QTLs, *qLP-CH24-D3-1*, *qLP-CH25-D12-1*, and *qLP-17-CH14-A8-1*, derived from NC05AZ06 (Zhang Z et al., 2019). Additionally, 26 QTLs on 9 chromosomes were reported from a cross of *G. hirsutum* × *G. barbadense*, including 9 stable or common QTLs that can be used in MAS (Shi et al., 2015). Currently, more than 60 stable major QTLs have been reported for LP and can be used in MAS breeding (Table 1 and 2; Supplementary Table S4).

**TABLE 2:** Some stable, major effective QTLs for LP which can be used in MAS.

QTL	Chromosome	Genetic position (cM)	LOD	*R* ^*2*^ (%)
*qLP-3-1*	Chr03	26.54	3.20	20.10
*qLP-A3-1*	Chr03	14.01	4.59	11.14
*qLP-A3-1*	Chr03	22.90	7.37	17.48
*qLP-A3-2*	Chr03	46.16	3.89	10.90
*qLP06.1*	Chr06	63.20	4.70	8.60
*qLP07.1*	Chr07	7.80	20.90	36.70
*qLP-A11-1*	Chr11	41.22	3.18	6.27
*qLP12.1*	Chr12	70.00	58.20	63.40
*qLP-15-1*	Chr15	24.41	7.35	19.86
*qLP-15-1*	Chr15	9.21	9.61	6.90
*qLP-15-2*	Chr15	33.25	6.35	18.47
*qLP-D2-1*	Chr15	0.01	4.15	8.69
*qLP-C16-6*	Chr16	177.70	4.39	10.11
*qLP-C16-7*	Chr16	189.30	5.79	11.60
*qLP-C17-1*	Chr17	91.30	10.81	27.51
*qLP-C17-2*	Chr17	94.90	11.70	26.40
*qLP21.1*	Chr21	165.60	15.60	24.30
*qLP-23-1*	Chr23	3.00	3.71	6.99
*qLP-D6-1*	Chr25	146.96	4.52	8.23
*qLP26.1*	Chr26	49.50	3.90	8.00

### High-Density Association Maps and QTNs of Cotton LP

A QTN is a marker associated with a QTL/gene and is likely in a candidate gene of the associated QTL if it is in a functional gene region. A QTN links a QTL with a nucleotide mutation. Different genome-wide association study (GWAS), single locus-GWAS (SL-GWAS), multi-locus GWAS (ML-GWAS), and restricted two-stage, multi-locus, multi-allele GWAS (RTM-GWAS) approaches have been used to search QTNs for LP in a large number of cotton accessions (Supplementary Table S1). For example, using RTM-GWAS, 86 single-nucleotide polymorphism linkage disequilibrium block (SNPLDB) loci for LP were identified from 315 cotton accessions (Su J et al., 2020). A total of 719 upland cotton accessions were screened by GWAS combined with cottonSNP63K array. High density SNP maps have been constructed (Sun et al., 2018), including more than 16 association maps (Supplementary Table S1). Many candidate genes for agronomic traits have also been reported (Fang et al., 2017; Huang et al., 2017; Sun et al., 2018; Shen et al., 2019; Su et al., 2019; Su J et al., 2020; Zhu G et al., 2020; Yu et al., 2021).

Using GWAS, six significant SNPs associated with LP were identified on chromosomes A02, A04, D03, and D12, together explaining approximately 43.41% of the total phenotypic variation. Three loci, *i03389Gh*, *i33922Gh*, and *i03391Gh*, are near each other on chromosome D03 (Sun et al., 2018). A comprehensive genomic assessment of modern improved upland cotton was performed based on the genome-wide re-sequencing of 318 landraces and modern improved cultivars or lines. Two major haplotypes with two nonsynonymous SNPs are associated with higher LP and more bolls per plant (*GhLYI-A02* ^*HLB*^), and lower LP and fewer bolls per plant (*GhLYI-A02* ^*LLB*^) (Fang et al., 2017). A diversity panel consisting of 355 upland cotton accessions was subjected to specific-locus amplified fragment sequencing (SLAF-seq). Twelve SNPs associated with LP were detected via GWAS, with five SNP loci distributed on chromosomes A_t_3 (A02) and A_t_4 (A08) that contain two major effective QTLs that were detected in best linear unbiased predictions (BLUPs) and in more than three environments simultaneously (Su et al., 2016). To date, more than 1396 QTNs for LP have been reported (Supplementary Table S1).

### A Consensus Physical Map of QTLs for LP in Upland Cotton

**FIGURE 1:**
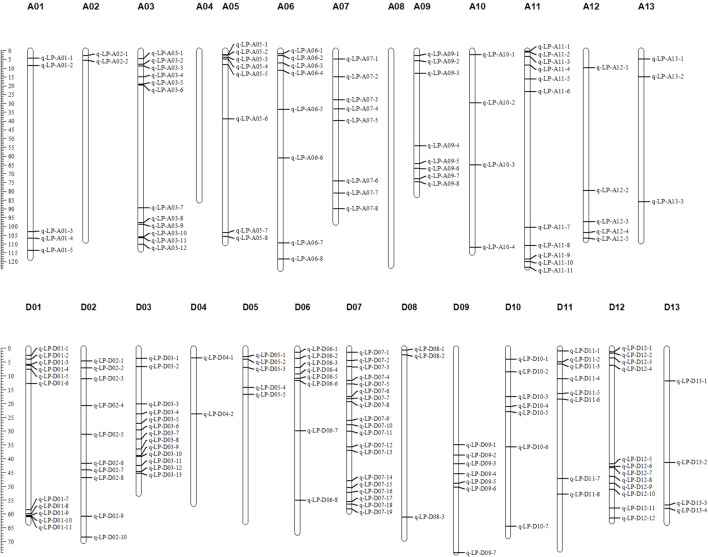
A consensus physical map of QTLs for LP in upland cotton. The QTLs were mapped to *G. hirsutum* genome by the locations of their linked markers. Left: the marker unit is Mb.

QTLs of LP represent genes controlling the phenotype of LP. QTNs are markers of the QTLs. Referring to the cotton genome sequences of *G. hirsutum* and *G. barbadense* (Li et al., 2015; Liu X et al., 2015; Yuan et al., 2015; Zhang et al., 2015; Huang et al., 2020), the QTLs of LP can be mapped using the locations of the associated QTNs or markers. In this way, more than 1439 QTLs for LP have been reported via genetic linkage maps. Excluding the replicate QTLs, there are more than 417 unique QTLs for LP. QTLs (183) with available tightly linked marker sequences were mapped to a consensus physical map of upland cotton (Figure 1). Obviously, many QTLs are clustered.

## Candidate Genes and Regulatory Network of Lint Percentage

### Candidate Genes of Cotton LP QTLs

Genes control phenotypes and isolation of the genes regulating LP is essential for functional gene marker development for MAS and gene engineering breeding. Fine mapping of the major effective QTLs for LP makes it possible to clone the candidate genes by map-based cloning. The availability of whole-genome sequences also paves a way to identify and clone functional genes, e.g., those relating to fiber development and increased LP (Pan et al., 2020; Sarfraz et al., 2021).

A diversity panel of 355 upland cotton accessions was subjected to specific-locus amplified fragment sequencing (SLAF-seq). Twelve SNPs associated with LP were detected via GWAS, in which five SNP loci distribute on chromosomes A_t_3 (A02) and A_t_4 (A08). *G. hirsutum_A02G1268* may determine LP (Su et al., 2016). A GWAS uncovered 23 polymorphic SNPs and 15 QTLs significantly associated with LP and identified two candidate genes, *G. hirsutum_D05G0313* and *G. hirsutum_D05G1124*, as promising regulators of LP (Song et al., 2019). Two ethylene-pathway-related genes are associated with increasing lint yield in improved cultivars, with two QTLs for LP, *qLP-5-17.8* and *Cs9_PF_21*, located within the two association loci, respectively. *G. hirsutum_A02G1392* is a homolog of the *AP2*/ethylene response factor (ERF)-type transcription-factor-encoding gene AINTEGUMENTA-like 6 (*AIL6*) in *Arabidopsis thaliana* (Fang et al., 2017). Another candidate gene, *G. hirsutum_D08G2376*, may control LP (Huang et al., 2017). Two genes, *G. hirsutum_D03G1064* and *G. hirsutum_D12G2354,* increase lint yield and with *G. hirsutum_D03G1067* are candidate genes of LP (Sun et al., 2018). Cotton *PROTODERMAL FACTOR1* (*GbPDF1*) gene is related with LP (Deng et al., 2012). A non-synonymous SNP (A-to-G) site in a gene encoding the cell wall-associated receptor-like kinase 3 (GhWAKL3) protein is highly correlated with increased LP (Ma et al., 2019). A class I TCP transcription factor (designated GbTCP), from *G. barbadense* affects LP (Hao et al., 2012). A cotton *GhH*_*2*_ *A*_*12*_ gene encoding a typical SPKK motif in the carboxyterminal and a plant-unique peptide-binding A/T-rich DNA region is involved in fiber differentiation (Hao et al., 2014). Candidate gene *Ghir_A03G020290* of a stable QTL *qLPA03-1* for LP encodes a thioredoxin domain-containing protein 9 homolog (Gu et al., 2020). *G. hirsutum_D03G0919* (*GhCOBL4*), *G. hirsutum_D09G1659* (*GhMYB4*), and *G. hirsutum_D09G1690* (*GhMYB85*) of LP QTLs were identified from RILs with *G. barbadense* introgressions (Wang et al., 2020). A MYB gene (*GhMYB103*), with two SNP variations in *cis*-regulatory and coding regions, is significantly correlated with LP, implying a crucial role in lint yield (Zhu G et al., 2020). A gene orthologous to *Arabidopsis* receptor-like protein kinase HERK 1 (*GB_A07G1034*) is predicated as a candidate gene for LP improvement (Yu et al., 2021). Two NET genes, *GH_A08G0716* and *GH_A08G0783*, in novel QTL hotspots (*qtl24* and *qtl25*), are mainly expressed during early fiber development stages and exhibit significant correlation with LP (Zhu et al., 2021). More than 90 candidate genes of QTLs have been reported (Supplementary Table S5).

**FIGURE 2:**
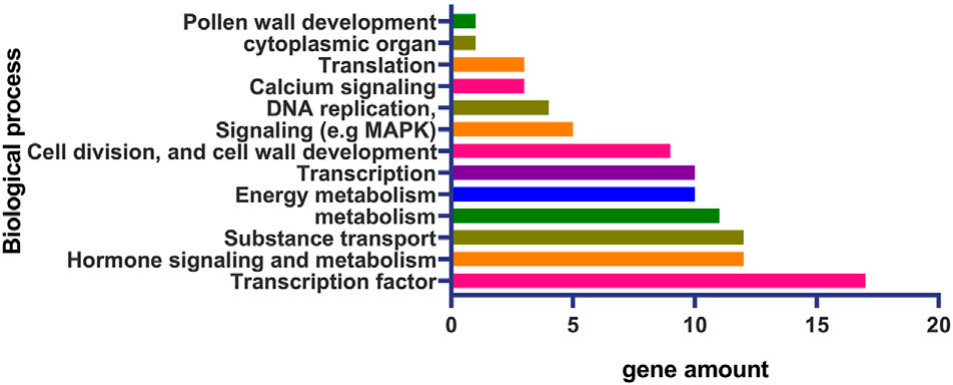
The major biological processes of the candidate genes of the QTLs for LP.

None of the genes of the major QTLs for LP have been isolated via map-based cloning, however, high density genetic linkage maps and multi-genome sequence data identified more than 90 candidate genes of QTLs for LP (Supplementary Table S5). According to the putative protein functions, most candidate genes of QTLs for LP are transcription factor (TF) and transcription related genes, with many candidate genes belonging to the MYB family of TF. According to the predicted gene functions of the candidate genes, transcription; signaling pathways including hormone, calcium (Ca^2+^), and MAPK; energy metabolism; substance transport; cell division; and cell wall development are important biological processes for LP formation (Figure 2; Table 3; Supplementary Table S5).

**TABLE 3:** The major biological processes related to the QTLs for LP.

Biological process	The candidate genes (homologs) of the QTLs for LP
Hormone signaling and metabolism	ET: *AIL6 (AP2), EIN3*; BR: *BZR1, LUT2*; JA: *TIFY10A*; IAA: *T85*; CTK: *MPK4*; ABA: *MYB52, KEG, PAA1, NET1D, NET3A*
Calcium signaling	*CPK17, CRCK2, CIPK6*
Signaling (e.g., MAPK)	*ASK8, PPC4-2, At1g12280, HERK1, Gpr107*
Transcription factor	*ZHD1, GhMYB4, GhMYB25, GhMYB25l, MYB52, GhMYB85, MYB86, GhMYB103, GbTCP, HDG11, GhHD-1, GhHD1, GhHOX1, VRN1, MYB308, ODO1, TTG1*
DNA replication	*PAA1, GhAATF1, bsdc1, GhmTERF1*
Transcription	*FRI, Bicc1, rpc11, At5g12190, SPP, SEN1, GhAATF1, GhmTERF1, GhH2A12, H2A*
Translation	*RPS10A, asnS, RPS1*
Cell division and cell wall development	*MPK4, FLA12, NCD80, At3g43860, CAP1, GhWAKL3, GbPDF1, GhCOBL4, NDC80*
Substance transport	*PHO1, TSC10B, COPT5, PRA1B1, UDP-GALT2, PYRAB13050, Gpr107, xpr1, bro1, G. hirsutum_D13G0342, TMN1, PLT5*
Energy metabolism	*APL2, COX5B-2, CRR21, WAXY, At3g43860, G. hirsutum_D03G1407, Acot9, GhGRAM31, GRAM35, GRAM5*
Pollen wall development	*TKPR1*
Metabolism	*INO1, RHA2A, CYP78A7, bsdc1, DTX33, YLS8, ROD1, UGT80A2, RAB71, TXND9, GEML5*
Cytoplasmic organ	*ATJ20*

Note: ET, ethylene; BR, brassinosteroid; JA, jasmonate acid; IAA, auxin; CTK, cytokinesis; ABA, abscisic acid. The detailed information of the reported genes is listed in Supplementary Table S5.

**FIGURE 3:**
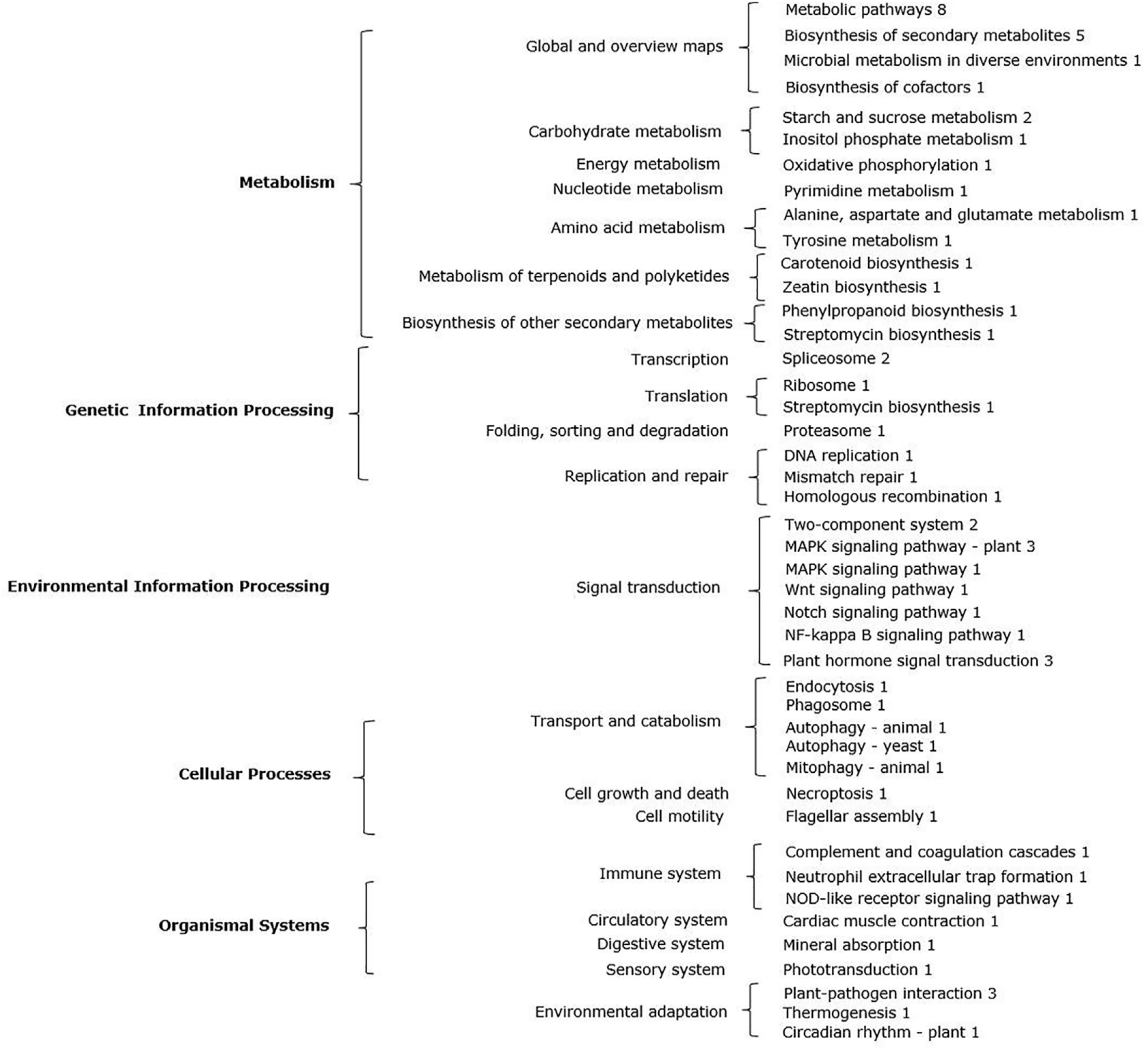
Candidate genes (gene number) that regulate LP are involved in major metabolic pathways.

To identify the major metabolic pathways of the candidate genes, pathway enrichment analysis was carried out using the Kyoto Encyclopedia of Genes and Genomes (KEGG; https://www.kegg.jp/kegg/) database. KEGG annotation analysis indicated that the 64 candidate genes of the QTLs for LP belonged to 44 KEGG pathways in *G. hirsutum* (Supplementary Tables S6 and S7). Among them, metabolic pathways, biosynthesis of secondary metabolites, MAPK signaling pathway, plant hormone signal transduction, plant-pathogen interaction, starch and sucrose metabolism, spliceosome, and two-component systems were most enriched (Table 4; Figure 3). The above results indicated that most of the candidate genes that affect LP in upland cotton do so through various metabolic pathways.

**TABLE 4:** Major metabolic pathways related to LP in cotton.

KEGG pathway	Ko Id	Candidate gene number
Metabolic pathways	Ko 01100	8
Biosynthesis of secondary metabolites	ko 01110	5
MAPK signaling pathway—plant	ko 04016	3
Plant hormone signal transduction	ko 04075	3
Plant-pathogen interaction	ko 04626	3
Starch and sucrose metabolism	ko 00500	2
Spliceosome	ko 03040	2
Two-component system	ko 02020	2

### Molecular Regulatory Network of Cotton Fiber Development

The coat of normal cottonseed is covered with lint and fuzz. The spinnable long fibers are called lint (25–35 mm) and initiate around flowering and then elongate rapidly afterward; the short fibers are called fuzz (∼5 mm), which develops at a later stage (Stewart, 1975; Du et al., 2001). Anatomically, cotton fibers are single-celled trichomes that differentiate from the outermost cell layer (protoderm) of the ovule. Cotton fiber is a highly elongated single cell, and its development synchronizes with and depends on seed development (Liu D et al., 2015). Cotton fiber development is a highly programmed and regulated process that can be divided into four discrete, yet overlapping stages on the basis of morphological characteristics during a 50-d period: initiation (0-3 days post anthesis, DPA), elongation (3-20 DPA), secondary cell wall thickening (16-40 DPA), and maturation (40-50 DPA) (Rong et al., 2005; Liu D et al., 2015; Qin et al., 2019). LP is a representative phenotypic trait of fiber development and the candidate genes of QTLs for LP (Table 3; Supplementary Tables S5 and S6) suggest relationships between LP and fiber development.

Many candidate genes of QTLs for LP participate in the regulation of fiber cell development. One candidate gene of QTLs for LP, *G. hirsutum_A02G1268* (*MIPS*), is highly expressed during the early fiber development stage and may determine LP by regulating seed and fiber development (Su et al., 2016). Two candidate genes of QTLs for LP, *G. hirsutum_D05G0313* and *G. hirsutum_D05G1124*, are highly expressed during ovule and fiber development stages (Song et al., 2019). A core genomic segment has a non-synonymous SNP (A-to-G) site in a gene encoding the cell wall-associated receptor-like kinase 3 (*GhWAKL3*), which may be involved in signaling and fiber cell wall-development to increase LP (Ma et al., 2019). Analysis of CSILs of SL15 (low-LP) and LMY22 (high-LP) indicate that delayed secondary-cell-wall thickening results in altered LP (Gai et al., 2007). In selective sweeps, an apoptosis-antagonizing transcription factor gene (*GhAATF1*) and mitochondrial transcription termination factor family protein gene (*GhmTERF1*) were found to be highly involved in the determination of LP (Han et al., 2020). Most of these genes are preferentially expressed at the initiation of cotton fiber and early elongation stages (Table 3; Supplementary Table S5).

**FIGURE 4:**
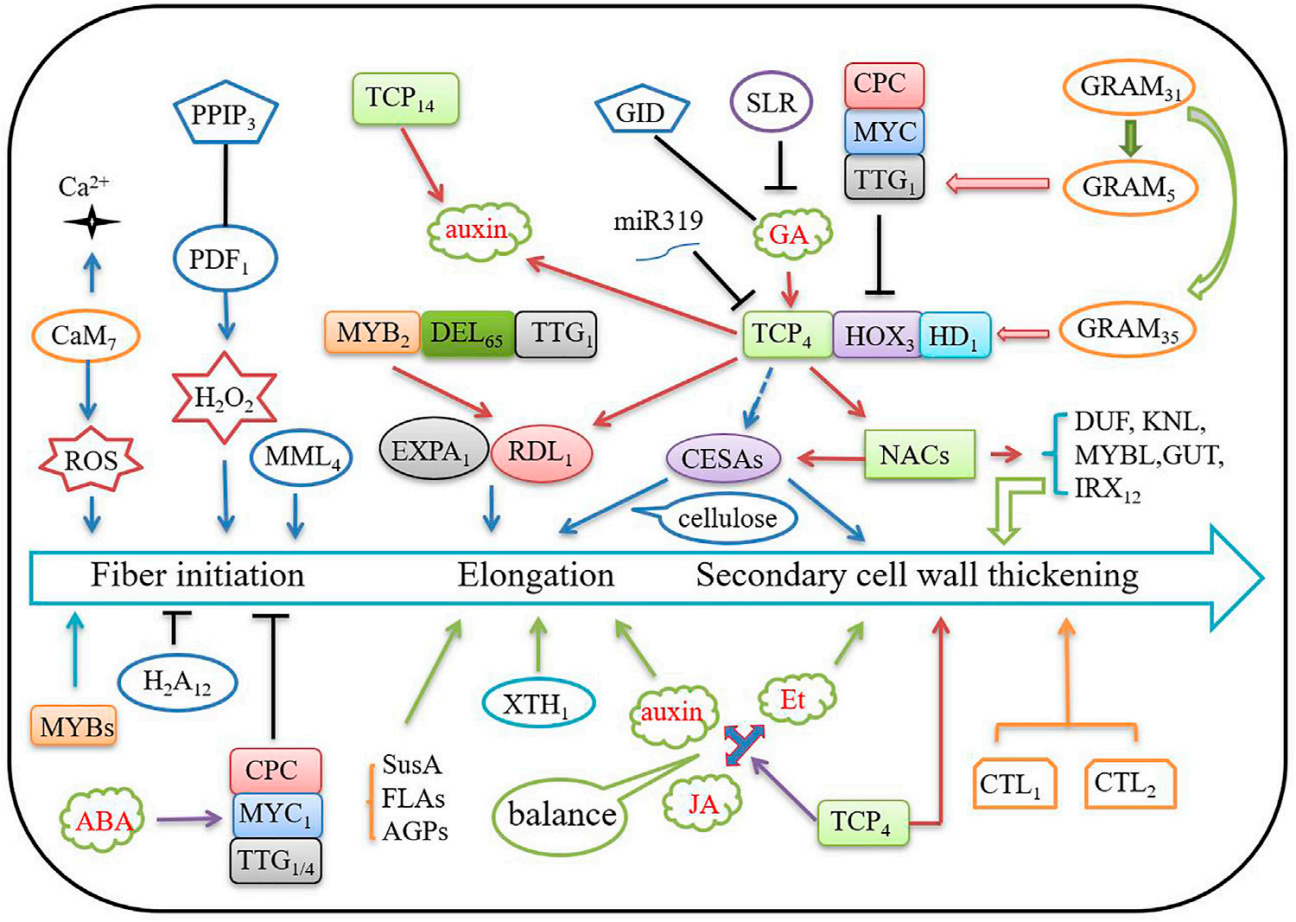
A molecular regulatory network of cotton fiber development. In the schematic, arrows indicate positive regulation; symbols of ‘T’ indicate negative regulation; lines indicate receptor or interaction; three tightly connected rectangles indicate tri-molecular complexes; red letters in clouds indicate phytohormones. Et, ethylene; ABA, abscisic acid; GA, gibberellin; JA, jasmonic acid; ROS, reactive oxygen species. “Gh” before gene symbols was omitted for space. The detailed information of the genes and their functions in cotton fiber development is listed in Supplementary Table S8.

Transgenic and functional gene assays have demonstrated that some genes that regulate fiber development can increase LP. *GbPDF1* is expressed preferentially during fiber initiation and early elongation. GbPDF1 plays a critical role together with interaction partners in hydrogen peroxide homeostasis and steady biosynthesis of ethylene and pectin during fiber development via the core *cis*-element HDZIP2ATATHB2 (Deng et al., 2012). *GbTCP* is preferentially expressed in the elongating cotton fiber from 5 to 15 DPA and GbTCP positively regulates the level of jasmonic acid (JA) to activate down-stream genes necessary for fiber elongation (reactive oxygen species, calcium signaling, ethylene biosynthesis and response, and several NAC [for NAM, ATAF1/2, and CUC2] and WRKY transcription factors) (Hao et al., 2012). *GhH*_*2*_ *A*_*12*_ is preferentially expressed during cotton fiber initiation and early elongation stages, suggesting a role in fiber differentiation (Hao et al., 2014). GhFSN1 is a cotton NAC transcription factor that acts as a positive regulator to control secondary cell wall (SCW) formation of cotton fibers by activating downstream SCW-related genes, including *GhDUF231L1*, *GhKNL1*, *GhMYBL1*, *GhGUT1,* and *GhIRX12* (Zhang et al., 2018). Overexpression of *GhCIPK6a* enhances expression levels of co-expressed genes induced by salt stress that scavenge reactive oxygen species (ROS), are involved in MAPK signaling pathways, and increase LP under salt stress (Su Y et al., 2020). MiR319, GhTCP4, and GhHOX3 dynamically coordinate the regulation of fiber elongation (Cao et al., 2020). The glucosyltransferases, Rab-like GTPase activators, and myotubularins (GRAM) domain gene *GhGRAM31* (*Ghir_D02G018120*) regulate fiber elongation and affect LP. GhGRAM31 directly interacts with GhGRAM5 and GhGRAM35. GhGRAM5 also interacts with the transcription factor GhTTG1, while GhGRAM35 interacts with the transcription factors GhHOX1 and GhHD1 (Ye et al., 2021). GhMYB109 is required for cotton fiber development (Pu et al., 2008). Overexpression of GhMYB3 in cotton leads to a slight increase in LP (Jiang et al., 2021). Over-expressing TF genes, *GhHD-1*, *GhMYB25*, and *GhMYB25*-like, in cotton can increase the density of fiber and fiber yield under field conditions. There are positive relationships between lint yield and LP and lint yield and fiber density in transgenic lines (Liu et al., 2020). Currently, more than 50 genes related to fiber development have been reported, including the *miR319* gene (Supplementary Table S8). The MYB-bHLH-WD40 (including MYB-DEL-TTG; CPC-MYC-TTG) (Liu D et al., 2015; Shangguan et al., 2021) and TCP-HOX-HD (Cao et al., 2020; Ye et al., 2021) regulatory complexes play key roles in cotton fiber development. The NAC-MYB-CESAs module is a central pathway of SCW cellulose biosynthesis (Cao et al., 2020). Phytohormone balance, Ca^2+^ signaling, and ROS also play key roles in the regulation of fiber development to affect cotton LP (Tang et al., 2014; Cheng et al., 2016) (Figure 4).

Transcriptomics studies have discovered many candidate genes and noncoding RNAs regulating LP. Cotton fibers initiate from the ovule *epidermis* on the day of anthesis. After fiber initiation, fiber cells expand rapidly as a result of increased intracellular swelling pressure and cell wall relaxation (Li et al., 2012; Qin et al., 2019). Comparative transcriptomics studies exploring differences in gene expression between long- and short-fiber cotton lines at five developmental stages identified 22 consistently down-regulated differentially expressed genes (DEGs) involved in fiber initiation and 31 consistently up-regulated genes involved in fiber elongation. These may be good candidate genes for improving LP. Of the candidate genes, ERF1 is involved in ethylene metabolism and is expressed at a higher level in long-fiber cotton lines at fiber initiation; TUA2 and TUB1 are involved in microtubule synthesis and expressed at a higher level in long-fiber cotton lines at fiber elongation; and *PER64* encodes a peroxidase (POD) and is expressed at a higher level in short-fiber cotton lines (Qin et al., 2019). Three lines with different LP were developed by crossing Xu142 with its fiberless mutant, Xu142 fl, and three long noncoding RNAs (lncRNAs) were identified as involved in fiber development. Silencing XLOC_545639 and XLOC_039050 in Xu142 fl increases the number of initiating fibers on the ovules, but silencing XLOC_079089 in Xu142 results in a short fiber phenotype (Hu et al., 2018). This demonstrated that lncRNAs manipulated the initiation of lint and fuzz fibers and affected LP. There is good evidence for the involvement of at least 62 genes, 45 miRANs, and 3 lncRNAs, which has been reported (Supplementary Table S9). Because the numbers of involved genes and potentially involved genes are exceptionally large, further work is required to determine the exact functions of these genes or miRNAs and the importance for LP development.

### Molecular Regulatory Network of Cotton LP

Although there have been many studies about cotton LP, the molecular mechanism of LP development remains largely unknown. The number of initial fibers determines the LP. The isolated candidate genes of QTLs for LP and the functionally analyzed genes suggest that key genes involved in fiber initiation and early elongation are the more important regulators, particularly those involved in hormone signaling, transcription, cell wall development, and signal transduction, as described above (Figure 4; Tables 3 and 4; Supplementary Tables S5, S6, S7, and S8).

**FIGURE 5:**
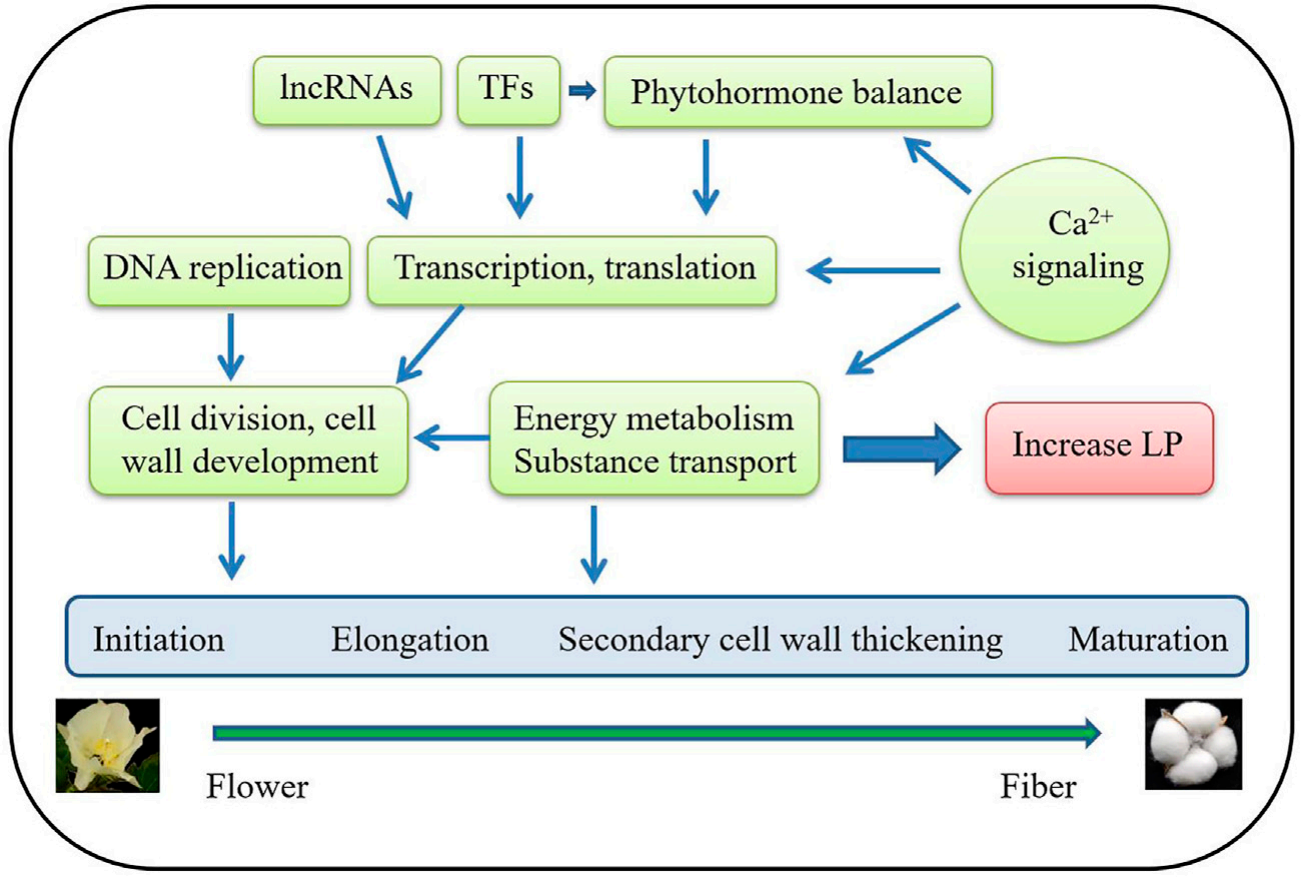
Proposed molecular regulatory network for LP. The gray rectangle indicates the four overlapped fiber developmental stages. The arrows indicate the regulatory relationships. The yellow rectangles indicate biological processes. The circles indicate different regulators. All genes and favorable alleles are preferentially and highly expressed during the initiation and early elongation stages; however, BR and JA pathways are repressed. The red rectangle indicates the active biological processes that help increase LP.

Based on the above summarized data, we proposed a molecular regulatory network for LP (Figure 5). Calcium signaling regulates hormone signaling, such as ABA signaling in leaf guard cells, and regulates gene expression, energy, and substance transport (McAinsh et al., 1997). TFs modulate transcription and hormone signaling and can affect LP. For example, MYB52 is involved in ABA signaling (Su J et al., 2020) and AIL6, a AP2-like ethylene-responsive TF, involved in ethylene (ET) signaling (Liu et al., 2014).

Most candidate genes of QTLs for LP (27/90) are TFs or otherwise related to transcription, suggesting that gene expression is more active during fiber initiation and elongation. The second most genes (12/90) are related to hormone signaling, indicating key roles in regulating fiber development (Table 3). The biological processes of signaling, cell division, cell wall development, energy metabolism, and substance transport are important for LP formation (Deng et al., 2012; Ma et al., 2019; Qin et al., 2019). In addition, some lncRNAs are involved in fiber development, and increase LP (Hu et al., 2018). According to the currently available data, longer fiber initiation and elongation stages should result in higher LP.

## Conclusion and Perspective

The current understanding of inheritance, QTLs, and candidate genes of LP is systemically summarized here. A large amount of genetic research results has been reported, including more than 417 unique QTLs for LP, 60 major effective QTLs, and more than 90 candidate genes of the QTLs. Additionally, more than 50 genes related to fiber development and microRNAs and long noncoding RNAs affecting LP have been reported. Most LP QTL candidate genes and genes related to fiber development affecting LP are preferentially expressed during fiber initiation and elongation. The biological processes of signaling, transcription, cell division, cell wall development, energy metabolism, and substance transport are important for LP development. By summarizing and analyzing the available data, this work reports the current state of the field and reveals what is known about cotton LP formation.

Although great genetic achievements have been made in cotton LP studies, it is difficult to improve LP without altering other yield components. The main reasons are as follows: (1) the inheritance of LP is complex; (2) LP is significantly negatively correlated with other key yield and quality components; (3) most QTLs for LP are unstable in different populations and environments, and may explain too little phenotype variance to trace with MAS; (4) LP has a significant negative heterosis value; (5) most genes of QTLs for LP are unknown and the molecular regulatory mechanism of LP is unclear. To address the above problems, future studies should: (1) Fully characterize the orchestrated inheritance model of LP and the relationship of LP with other yield and quality components; (2) identify more stable and major effective QTLs for LP; (3) improve elite germplasm resources of LP by breaking the linkage between LP and other deleterious yield and quality component QTLs/alleles; (4) study the molecular mechanisms of fiber development and increasing LP using omics and gene functional validation; and (5) develop effective techniques to pyramid favorable genes or alleles for high yield and high LP cotton breeding.
